# Follicular Lymphoma Evaluation Index (FLEX): A new clinical prognostic model that is superior to existing risk scores for predicting progression‐free survival and early treatment failure after frontline immunochemotherapy

**DOI:** 10.1002/ajh.25973

**Published:** 2020-09-16

**Authors:** Farheen Mir, Federico Mattiello, Andrew Grigg, Michael Herold, Wolfgang Hiddemann, Robert Marcus, John F. Seymour, Christopher R. Bolen, Andrea Knapp, Tina Nielsen, Carla Casulo

**Affiliations:** ^1^ The Royal Marsden NHS Trust Foundation London UK; ^2^ F. Hoffmann‐La Roche Ltd Basel Switzerland; ^3^ Austin Hospital Melbourne Victoria Australia; ^4^ HELIOS‐Klinikum Erfurt Erfurt Germany; ^5^ University Hospital Munich Munich Germany; ^6^ HCA Healthcare London UK; ^7^ Peter MacCallum Cancer Centre Royal Melbourne Hospital and University of Melbourne Melbourne Victoria Australia; ^8^ Genentech, Inc South San Francisco California USA; ^9^ Wilmot Cancer Institute University of Rochester New York New York USA

## Abstract

Patients with advanced‐stage follicular lymphoma (FL) who progress early after receiving first‐line therapy have poor overall survival (OS). Currently applied clinical prognostic models such as FL International Prognostic Index [FLIPI], FLIPI‐2 and PRIMA‐Prognostic Index [PRIMA‐PI] have suboptimal sensitivity and specificity to predict this poor prognosis subgroup. The primary objective was to develop a novel prognostic model, the FL Evaluation Index (FLEX) score, to identify high‐risk patients and compare its performance with FLIPI, FLIPI‐2 and PRIMA‐PI. Progression‐free survival (PFS) after first‐line immunochemotherapy was the key endpoint, while OS and progression of disease within 24 months (POD24) were also assessed. The model, which includes nine clinical variables, was developed using a cohort of patients with previously untreated advanced‐stage FL from the phase 3 GALLIUM trial (NCT01332968). The performance of the model was validated using data from the SABRINA trial (NCT01200758). In GALLIUM (n = 1004; 127 with and 877 without POD24), FLEX increased the intergroup (low‐risk/high‐risk) difference in 2‐year and 3‐year PFS rates and demonstrated superior intergroup differences in 2‐year and 3‐year OS rates compared with FLIPI, FLIPI‐2 and PRIMA‐PI. Sensitivity for a high‐risk score to predict POD24 was 60% using FLEX compared with 53% for FLIPI and FLIPI‐2, and 69% for PRIMA‐PI, while specificity was 68% for FLEX compared with 58% for FLIPI, 59% for FLIPI‐2 and 48% for PRIMA‐PI. The prognostic value of FLEX in SABRINA was similar to FLIPI. Therefore, FLEX appears to perform better than existing prognostic models in previously untreated FL, in particular for the newer treatment regimens.

## 1 INTRODUCTION

Despite recent advances in immunochemotherapy, patients with advanced‐stage follicular lymphoma (FL) who experience early disease progression after first‐line therapy, in particular those with progression of disease within 24 months (POD24), represent a subgroup of high‐risk individuals with a particularly poor prognosis.^1^, ^2^, ^3^, ^4^, ^5^, ^6^, ^7^ It is important to be able to identify these high‐risk patients at diagnosis, prior to treatment, as they may be candidates for alternative, risk‐adapted therapies, which may include investigational regimens in clinical trials.^8^ While several clinical prognostic models, such as the Follicular Lymphoma International Prognostic Index (FLIPI) and FLIPI‐2, exist and are useful predictors of outcome,^9^, ^10^, ^11^ they have relatively low sensitivity for predicting disease progression.^5^, ^9^, ^12^ Furthermore, as these models were developed before the recent advances in therapy, their applicability to current immunochemotherapy regimens is unclear. Even the more recent PRIMA‐Prognostic Index (PRIMA‐PI; a simple score incorporating β_2_ microglobulin and bone marrow involvement) was developed from trial data where bendamustine and obinutuzumab were not included as treatment options.^13^

In addition to clinical models, several clinicogenomic scores have been developed in an attempt to optimize the identification of patients with high‐risk FL. These measures have important prognostic value but still have suboptimal sensitivity and/or specificity for POD24. For example, the m7‐FLIPI risk score, which integrates FLIPI with the Eastern Cooperative Oncology Group performance status (ECOG PS) and the mutation status of seven genes,^14^ demonstrated a 61% and 43% sensitivity for POD24 after first‐line immunochemotherapy in two independent patient cohorts.^12^ A simplified modification of m7‐FLIPI, known as the POD24‐PI, which integrates FLIPI and the mutation status of three genes, increased the sensitivity for POD24 (to 78% and 61% in the two cohorts), but at the expense of lower specificity.^12^ Clearly, both tools appear to be better at identifying high‐risk patients than existing clinical models,^12^ but their sensitivity/specificity for POD24 has not been optimized. They require complex genetic analysis (which is not easily accessible to most community hospitals) and neither has been developed in bendamustine‐ or obinutuzumab‐treated patients. A separate 23‐gene signature model developed by Huet et al.^15^ Using NanoString technology demonstrated low sensitivity for POD24 (43%), despite being able to predict progression‐free survival (PFS) independently of FLIPI and anti‐CD20 maintenance treatment.^15^ This model was also developed in patient cohorts not receiving obinutuzumab and bendamustine.

Here, we have developed a new prognostic model — the Follicular Lymphoma Evaluation Index (FLEX) — based solely on clinical variables (allowing ease of application in routine practice), that is able to identify high‐risk patients by predicting both PFS (key endpoint) and POD24 following first‐line immunochemotherapy for FL with current recommended regimens. The aim was to develop a simple prognostic model that is easy to interpret. In developing this model, we used patient‐level data from the phase 3 GALLIUM trial.^16^ This study was selected as it provides a large training dataset and incorporates current standard‐of‐care treatments, including obinutuzumab and bendamustine. An independent dataset from the phase 3 SABRINA trial, which utilized cyclophosphamide, doxorubicin, vincristine and prednisone (CHOP)‐based and cyclophosphamide, vincristine and prednisone (CVP)‐based treatments, was used to validate the model, although it should be noted that there was no bendamustine treatment arm in SABRINA.^17^ A preliminary version of the FLEX was presented at the 2018 American Society of Hematology Annual Meeting.^18^ Since this presentation, the two treatment modifiers, chemotherapy backbone and anti‐CD20 antibody, have been removed to make it more generally applicable; omission of these variables has not resulted in any loss in performance, among those considered.

## 2 METHODS

### 2.1 Training cohort

Data for the training cohort were derived from an updated efficacy analysis of all 1202 patients with FL enrolled in the randomized phase 3 GALLIUM trial (NCT01332968; cut‐off date, February 12, 2018; data snapshot, April 26, 2018; median follow‐up, 57 months).^16^, ^19^ Patients in GALLIUM were aged ≥18 years with previously untreated CD20‐positive FL (histologic grades 1‐3A), stage III/IV disease (or stage II with bulky disease) and an ECOG PS of ≤2.^16^ The full study design and inclusion/exclusion criteria are published elsewhere.^16^ In GALLIUM, patients were randomized 1:1 to receive 6‐8 cycles of obinutuzumab‐based or rituximab‐based induction immunochemotherapy, followed by maintenance with the same antibody for 2 years (or until disease progression or withdrawal) in responders. The chemotherapy backbone (CHOP, CVP or bendamustine) was selected upfront by each individual study center and was a stratification factor for the trial. The primary endpoint was investigator‐assessed PFS.

The study was conducted in accordance with the principles set out in the updated Declaration of Helsinki, the International Conference on Harmonisation Guideline for Good Clinical Practice and all applicable local laws and regulations. The study protocol and its amendments and other study‐related materials were approved by the institutional review boards/ethics committees at participating centers. Written informed consent was provided by all patients.

### 2.2 Model inputs

Based on data availability in GALLIUM and prior evidence of biological plausibility (ie, by a review of published data demonstrating an association with adverse outcomes and on the basis of clinical discussions), 17 clinical variables were identified as potential model inputs before initiating the analysis, and were included in the primary evaluation (Table S1). Continuous variables were dichotomized using established cut‐offs or, in the case of sum of the products of lesion diameters (SPD), by inputting separate variables for each quartile. Positron emission tomography (PET) imaging variables were not included as potential model input as PET scan results were only available for approximately half of all GALLIUM patients.

Of note, the impact of dichotomization of input variables has been assessed to be very small, in a sensitivity analysis (Figure S1), compared with using continuous variables that considerably increases complexity for the user.

Treatment variables were used for model fitting but were excluded from the final construct as the aim of the study was to establish a general prognostic model that performs well independently of treatment.

### 2.3 Validation cohort

The FLEX model was validated using an independent dataset from the SABRINA trial: a randomized phase 3 study (NCT01200758) investigating the pharmacokinetic non‐inferiority, efficacy and safety of subcutaneous vs intravenous rituximab plus 6‐8 cycles of chemotherapy, followed by rituximab maintenance for 2 years (in responders), for the first‐line treatment of patients with FL.^17^ Enrolled patients were aged ≥18 years with previously untreated CD20‐positive FL (histologic grades 1‐3A) and an ECOG PS of ≤2. Chemotherapy backbones in the SABRINA study were CVP (received by 37% of patients) or CHOP (received by 63% of patients); none of the patients in SABRINA received bendamustine.

### 2.4 Statistical analysis

The FLEX prognostic model was built using penalized multivariable Cox regression methodology with elastic net regularization.^20^ This method yields a choice of plausible models with a range of clinical variables, where cross‐validation errors vary depending on the values of the tuning parameters. The strength of clinical variables to predict prognosis was assessed based on hazard ratios (HRs), and 95% confidence intervals (CIs) for PFS in the training (GALLIUM) cohort. The model selected (lambda penalty parameter) was within one standard deviation of that with minimum cross‐validation error. All clinical variables included in the final model were given equal weight and a clinical score (termed the “FLEX score”) was calculated by summating the number of risk factors for each patient. The cut‐off on the receiver operating characteristic (ROC) curve for predicting PFS events vs censoring that was closest to the (0,1) point was used to categorize patients into low‐risk and high‐risk categories.

The performance of the FLEX score was tested by assessing the effect of risk categorization (high‐risk vs low‐risk) on PFS and overall survival (OS), and then comparing these data with those obtained using established prognostic indices (FLIPI, FLIPI‐2 and PRIMA‐PI). For each prognostic tool, intergroup differences in estimated 2‐year and 3‐year PFS and OS rates between patients classified as high‐ and low‐risk were calculated. All survival outcomes were estimated using Kaplan‐Meier methodology. To test the robustness of the FLEX model, sensitivity analyses were undertaken to assess the effect of chemotherapy backbone, exclusion of CVP‐treated patients (as CVP is used less often in current practice), additional risk stratification and increasing the cut‐point for defining high risk on PFS. Additionally, Cox proportional‐hazards analysis of PFS (based on HRs and 95% CIs) evaluated the effects of treatment regimen and the nine components of the FLEX score on the results. The effects of treatment regimens on the PFS data were also determined for FLIPI and PRIMA‐PI.

The sensitivity and specificity of a high‐risk score to predict POD24 were calculated for each prognostic tool, and were used as a measure of accuracy for predicting POD24, which was defined as progressive disease (PD) or death due to disease within 24 months of randomization (noPOD24 = no PD or lymphoma‐related death in that period).

In addition, we performed a time‐dependent area under the ROC curve (AUROC) analysis to have a more global assessment of discrimination ability of the different scores examined.

## 3 RESULTS

### 3.1 Prognostic model

Nine clinical variables (male sex, SPD in the highest quartile, histologic grade 3A, >2 extranodal sites, ECOG PS >1, hemoglobin <12 g/dL, β_2_ microglobulin >institutional upper limit of normal [ULN], peripheral blood absolute natural killer [NK] cell count <100/μL and serum lactate dehydrogenase [LDH] > ULN) were retained by the methodology and selected for inclusion in the final model based on their HRs for PFS (Table S2). Two variables each were adapted from FLIPI (hemoglobin and LDH) and FLIPI‐2 (β_2_ microglobulin and hemoglobin); novel variables included SPD, gender and NK cell count.

Patients were categorized as “low‐risk” if they scored 0‐2 (n = 645/1004 patients with complete data) or “high‐risk” if they scored 3‐9 (n = 359/1004 patients). The cutoff‐point of 3 for risk categorization reflected the number closest to the (0,1) point on both the ROC curves for predicting PFS events at the 3‐year landmark and POD24 event, in the training (GALLIUM) cohort. This cut‐point provided the best balance between true‐positives and false‐positives for POD24.

### 3.2 Model performance

**FIGURE 1:**
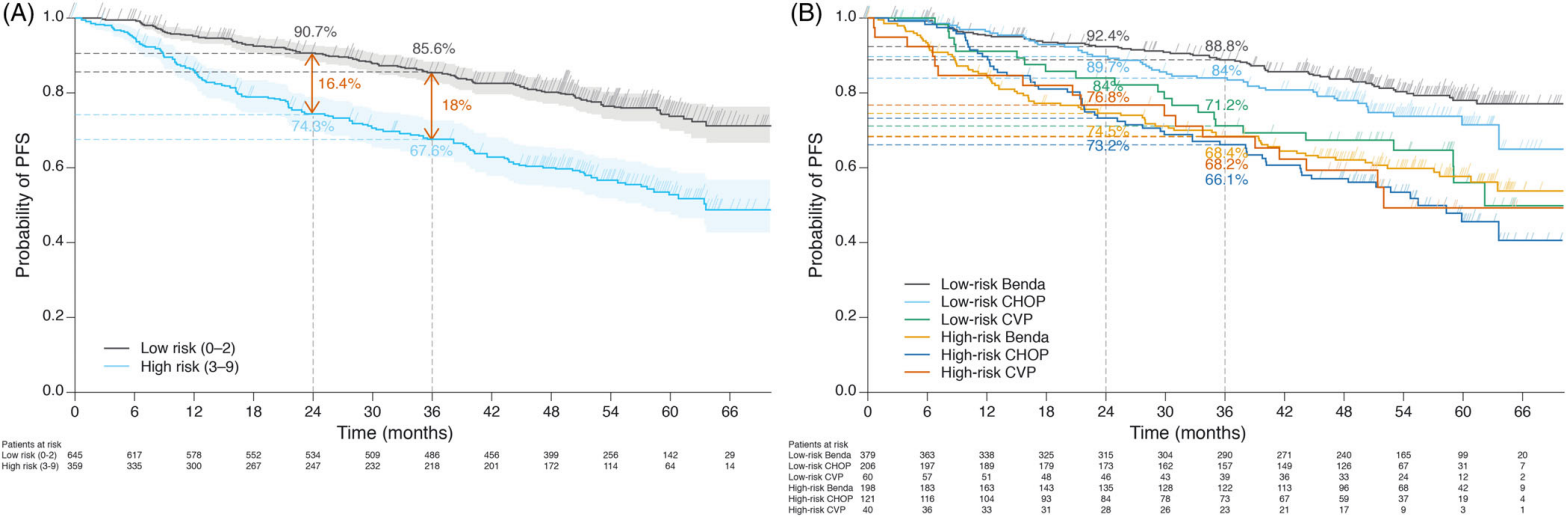
Progression‐free survival A, according to FLEX risk category (low and high risk) and B, by chemotherapy backbone in GALLIUM (probability of progression‐free survival at 2 and 3 years is shown for all chemotherapy subgroups). Benda, bendamustine; CHOP, cyclophosphamide, doxorubicin, vincristine, prednisone; CVP, cyclophosphamide, vincristine, prednisone; FLEX, Follicular Lymphoma Evaluation Index; PFS, progression‐free survival

The performance of the FLEX model was assessed in a training cohort of 1004 patients from the GALLIUM trial; these patients were those with complete data for all nine components of the FLEX score. Applying the established risk categorization to the GALLIUM data, 2‐year and 3‐year PFS rates of 91% and 86%, respectively, were observed in FLEX low‐risk patients compared with 74% and 68%, respectively, in high‐risk patients (Figure 1A). Two‐year and 3‐year OS rates were 98% and 97%, respectively, in low‐risk patients, and 90% and 87%, respectively, in high‐risk patients.

**FIGURE 2:**
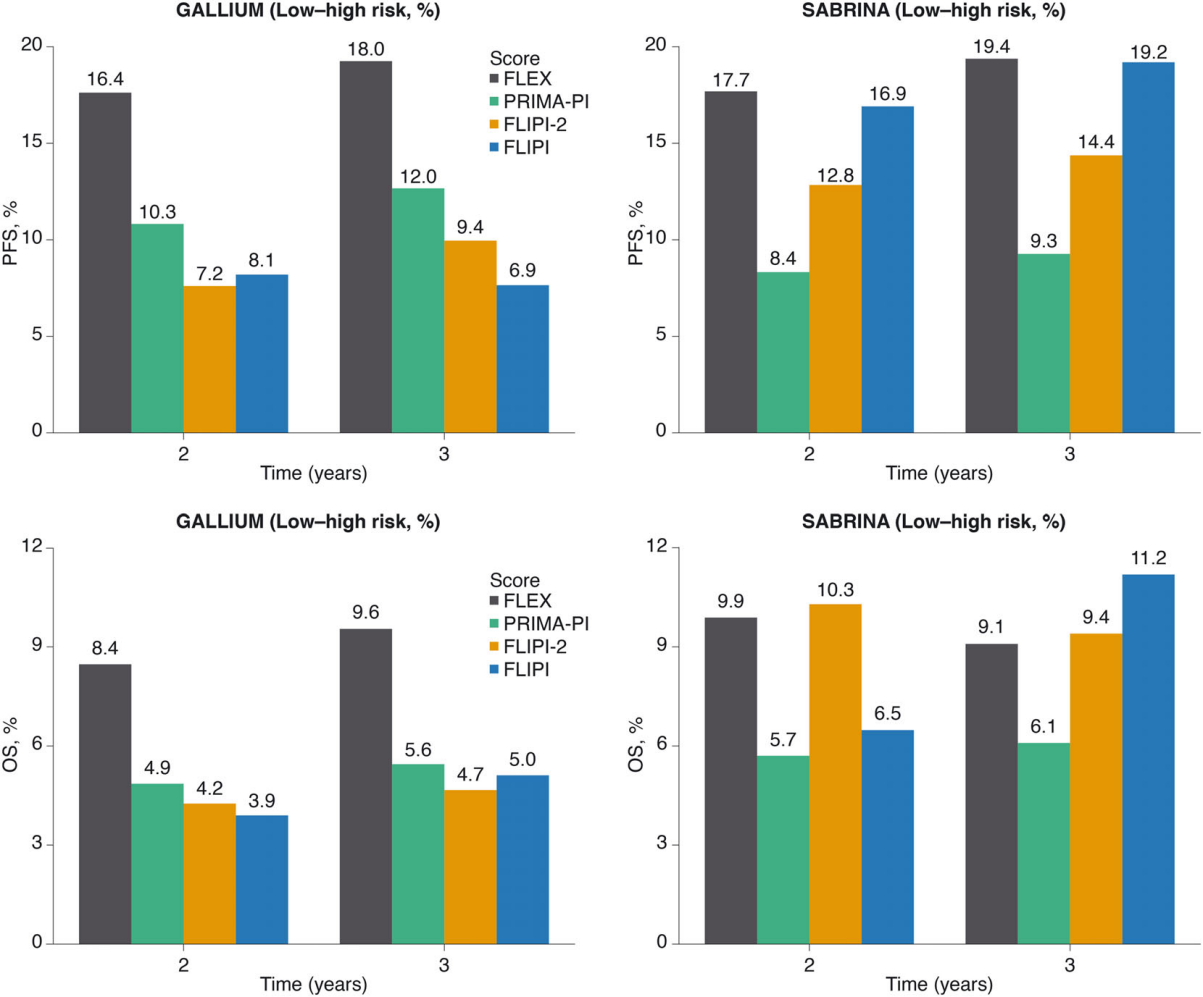
Intergroup differences in 2‐year and 3‐year progression‐free survival and overall survival between low‐risk and high‐risk patients in GALLIUM and SABRINA, defined using FLEX, PRIMA‐PI, FLIPI‐2 and FLIPI. FLEX, Follicular Lymphoma Evaluation Index; FLIPI, Follicular Lymphoma International Prognostic Index; OS, overall survival; PFS, progression‐free survival; PRIMA‐PI, PRIMA‐Prognostic Index

The intergroup (low‐risk to high‐risk) differences in 2‐year and 3‐year PFS rates in the training cohort (GALLIUM) were numerically higher with FLEX than with FLIPI, FLIPI‐2 or PRIMA‐PI (Figure 2). A similar observation was seen for the intergroup (low‐high risk) differences in 2‐year and 3‐year OS rates (Figure 2).

During model development, substituting SPD with the presence of bulky disease (defined as any lesion >7 cm diameter) resulted in a small decrease in the performance of the FLEX score (not shown) but, notably, additional less relevant variables like geographical region, which may be correlated with disease burden, were retained by the procedure. Without SPD or bulky disease the performance of the FLEX model for predicting prognosis was diminished relative to other clinical scores.

### 3.3 Sensitivity and additional analyses

We conducted various post‐hoc analyses to assess if the performance of the FLEX model was consistent. These included analysis in the various chemotherapy backbone groups (excluding patients receiving CVP), assessing the impact of an intermediate risk category and also the impact of using a higher cut‐off for “high‐risk” patients. The performance of the FLEX model for PFS was consistent across backbone chemotherapy groups, with the greatest separation in the PFS curves for low‐risk vs high‐risk patients observed among bendamustine‐treated patients (Figure 1B). Excluding CVP‐treated patients from the analysis had little effect on the performance of the FLEX model; the PFS curves for low‐risk vs high‐risk patients in this subpopulation were similar to those seen in the overall training cohort.

Splitting patients into three FLEX risk categories (low [score of 0‐1], intermediate [score of 2] and high‐risk [score of ≥3]), instead of two, provided additional risk stratification with 2‐year PFS rates of 93%, 88% and 74%, and 3‐year PFS rates of 88%, 82% and 67%, respectively. Using a higher cut‐point of ≥4 to define FLEX high‐risk instead of ≥3 (the optimal threshold) resulted in fewer high‐risk patients (n = 172; 17%), but increased the intergroup (low‐high risk) differences in 2‐year and 3‐year PFS rates to 24% and 25%, respectively.

Cox proportional‐hazards analysis of PFS showed that FLIPI and PRIMA‐PI had a lower prognostic value across treatment regimens than the FLEX score (Figure S2). Notably, FLIPI was not able to separate prognostic categories among patients who received obinutuzumab plus bendamustine. The Cox proportional‐hazards analysis of PFS for bendamustine‐treated vs CHOP/CVP‐treated patients and for rituximab‐treated vs obinutuzumab‐treated patients showed that the impact of the majority of FLEX score components were treatment independent. However, an exception was for NK cell count in both groups, and ECOG PS in bendamustine‐treated vs CHOP/CVP‐treated patients only (Figure S3), where a trend was observed. However, sample sizes were too small to draw clear conclusions, especially for ECOG PS.

As an additional sensitivity analysis, we assessed the impact of categorization performed on some of the variables in the initial search list. Figure S1 shows ROC curves on the POD24 event, comparing the linear predictors of the two Cox models: one with categorized variables, and the other with variables modeled as (possibly transformed) continuous (most notably log‐LDH and square root of SPD). From there it seems the impact of categorizing a few continuous variables was negligible in GALLIUM and in SABRINA, with the only exception being the lower end of the spectrum in SABRINA data (worse discrimination for low‐risk patients).

Moreover, the survival profile of patients with missing data on FLEX components appears similar to that of complete cases, with a PFS HR for “incomplete” vs “complete” FLEX data of 0.89 (95% CI: 0.68‐1.17; *P* = .40). From additional modeling of the propensity of missingness (not shown), we could only observe some geographical differences (eg, Asian patients had fewer missing data, CHOP patients from Western Europe had more missing data) and, more importantly, there was no apparent impact of missing vs non‐missing FLIPI data.

### 3.4 Sensitivity and specificity for POD24

**FIGURE 3:**
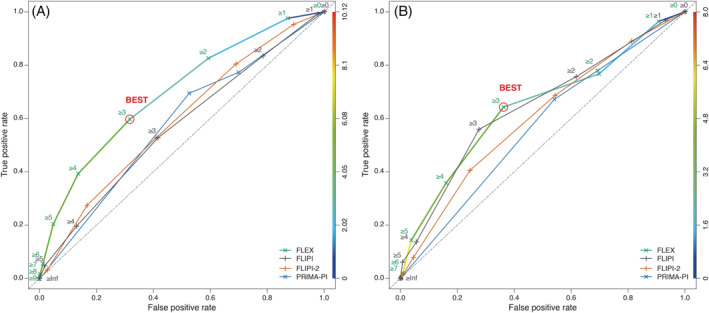
ROC curves for A, GALLIUM and B, SABRINA, comparing FLEX, FLIPI, FLIPI‐2 and PRIMA‐PI, for POD24; the selected cut‐off is highlighted. The right‐hand axis indicates the score values. FLEX, Follicular Lymphoma Evaluation Index; FLIPI, Follicular Lymphoma International Prognostic Index; POD24, progression or death due to the disease within 24 months of first‐line therapy; PRIMA‐PI, PRIMA‐Prognostic Index; ROC, receiver operating characteristic

**FIGURE 4:**
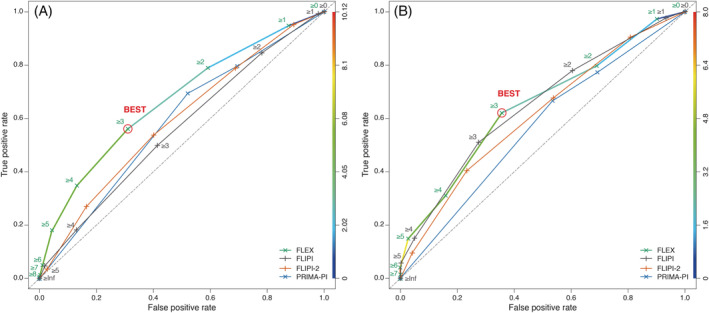
ROC curves for A, GALLIUM and B, SABRINA, comparing FLEX, FLIPI, FLIPI‐2 and PRIMA‐PI, for PFS36 event; the selected cut‐off is highlighted. FLEX, Follicular Lymphoma Evaluation Index; FLIPI, Follicular Lymphoma International Prognostic Index; PFS36, progression‐free survival within 36 months; PRIMA‐PI, PRIMA‐Prognostic Index; ROC, receiver operating characteristic

Sensitivity for a high‐risk score to predict POD24 in the training cohort was 60% with FLEX compared with 53% for FLIPI and FLIPI‐2, and 69% for PRIMA‐PI (based on 127 POD24 and 873 noPOD24 patients with complete data). Specificity for POD24 was 68% with FLEX compared with 59% for FLIPI and FLIPI‐2, and 47% for PRIMA‐PI (Figure 3). Using a higher cut‐point of ≥4 to define FLEX high‐risk patients reduced the sensitivity for POD24 whilst increasing specificity. The ROC curves for both POD24 events (Figure 3) and PFS events at the 3‐year landmark (Figure 4) show the discrimination ability of the proposed score.

### 3.5 Validation

When applied to the cohort of 342 evaluable patients from the SABRINA trial, the FLEX model was able to discriminate between those who were high‐ and low‐risk, as indicated by the Kaplan‐Meier estimates showing an intergroup difference of 18% and 19% in 2‐year and 3‐year PFS rates, respectively (Figure 2). However, while FLEX demonstrated numerically higher 2‐year and 3‐year PFS rates compared with FLIPI, FLIPI‐2 and PRIMA‐PI (albeit only marginally vs FLIPI; Figures S4 and S5), it was numerically lower than FLIPI or FLIPI‐2 for OS at 3 years (Figure 2). Consistent performance of the FLEX score components for PFS was demonstrated in both the GALLIUM and SABRINA studies (Figure S6).

These results are also confirmed by the time‐dependent AUROC analysis performed on both GALLIUM and SABRINA cohorts (Figure S7). FLEX performs similarly to FLIPI in SABRINA but better than other scores across time‐points, including notably better than PRIMA‐PI.

## 4 DISCUSSION

Various clinical and clinicogenomic scores are available for predicting prognosis in patients with previously untreated advanced‐stage FL who are about to commence first‐line therapy.^9^, ^10^, ^11^, ^12^, ^13^, ^14^, ^15^ However, the clinical utility of these models is limited by a lack of data on how they perform in patients treated with current frontline standard‐of‐care regimens, including obinutuzumab and bendamustine, and they are rarely used in everyday practice. They also demonstrate suboptimal sensitivity and specificity for predicting early relapse (eg, POD24) following first‐line immunochemotherapy for FL,^5^, ^9^, ^12^ which is an important indicator of outcome.^1^, ^2^, ^3^, ^4^, ^5^, ^6^, ^7^ Using a large dataset from the GALLIUM trial,^16^, ^19^ we have developed a new clinical prognostic model, FLEX, with easily measurable components, that has both greater accuracy for predicting POD24 (in terms of its combined sensitivity and specificity) than existing clinical tools (FLIPI, FLIPI‐2 and PRIMA‐PI) and is a robust indicator of PFS irrespective of first‐line treatment regimen. Importantly, FLEX is the first clinical score in FL to be developed in patients treated with bendamustine and either obinutuzumab or rituximab; it is therefore applicable to patients treated with the current standard of care.

In the training cohort from the GALLIUM study,^16^, ^19^ FLEX was better than FLIPI, FLIPI‐2 and PRIMA‐PI at discriminating between patients with a good or poor prognosis in terms of both PFS and OS. Also, FLEX demonstrated an improved ability to predict PFS compared with FLIPI‐2 or PRIMA‐PI in the validation (SABRINA^17^) cohort, and a comparable ability to FLIPI. The performance of FLEX, FLIPI and FLIPI‐2 were similar in their ability to predict OS in the validation cohort; for this endpoint, all three of these tools performed better than PRIMA‐PI. The different performance of FLEX in the GALLIUM and SABRINA cohorts may be due to differences in the design of the two studies, in terms of patient eligibility, and such reduction in performance is typical when moving from test to validation cohort applications.^16^, ^17^, ^19^ Nonetheless, regardless of which cohort was used for the assessment, FLEX was still able to consistently discriminate high‐risk vs low‐risk patients, particularly with respect to PFS; therefore the reliability of the FLEX score supports the robustness and stability of the FLEX score independent of treatment.

In addition to predicting PFS and OS outcome, FLEX demonstrated higher predictability for POD24 compared with both FLIPI and FLIPI‐2, and higher specificity for POD24 than FLIPI, FLIPI‐2 and PRIMA‐PI. In this study, FLIPI identified just over half of all patients with POD24 events. Although these results suggest that FLEX is a good clinical model for predicting early disease relapse and prognosis, its performance is still not optimal. Furthermore, individual patient preference and circumstances may also influence the treatment decision.

Ideally, physicians would like to use a prognostic score that is both easy to calculate and has variables that are readily available. Due to its simplicity, PRIMA‐PI (which utilizes just β_2_ microglobulin and bone marrow involvement) is a popular choice among physicians.^13^ However, in this study, PRIMA‐PI did not perform consistently between GALLIUM and SABRINA datasets. Based on our analyses, FLEX may be a better, albeit more elaborate, alternative clinical score for patients treated with current standard therapy. Although we acknowledge potential limitations due to categorization of some of the chosen variables the impact appeared negligible.

A key challenge when developing a new prognostic tool is to balance the complexity of the model with its added accuracy. Thus, SPD, which assesses tumor burden, represents the most complex clinical variable in the FLEX model. Importantly, during the model development, we found that SPD had one of the highest HRs for PFS; however, the reporting of SPD requires a radiologist to calculate the sum of the product diameters for up to six target lesions, which can be a time‐consuming process. In clinics where measurement of SPD is not possible, or is not routinely assessed, the presence of bulky disease may be an alternative and more accessible clinical variable, although its reliability compared with SPD remains to be confirmed.

The inclusion of NK cell count in the FLEX model likely reflects the involvement of NK cells as key effectors for anti‐CD20 antibodies, such as obinutuzumab and rituximab.^21^, ^22^ A recent study has shown that both low NK cell count and low tumor NK cell gene expression are surrogate markers of an impaired antitumor response to anti‐CD20 antibodies in both FL and diffuse large B‐cell lymphoma.^23^ In contrast to older clinical scores, variables such as age and bone marrow involvement were not retained in the FLEX model, and appear less relevant in the current era, at least across the age range enrolled in these clinical trials.

It may be argued that purely clinical prognostic scores are not optimal in the modern era of integrated clinicogenomic models. However, an analysis of two high‐risk gene expression signatures using RNA sequencing data from GALLIUM demonstrated that the prognostic ability of both genomic models was treatment dependent (ie, bendamustine‐treated vs CHOP/CVP‐treated patients behaved differently); genes that may be prognostic in one treatment subgroup may not therefore be prognostic in others.^24^ Consequently, it is not currently possible to create one clinicogenomic model applicable to all patients. Moreover, the potential benefits of incorporating genomic data into a clinical model might be outweighed by increased cost and complexity. In contrast, clinical models, such as FLEX, perform well irrespective of treatment received, providing broad applicability.

To conclude, in the GALLIUM study, our new clinical prognostic model, FLEX, was more accurate at discriminating patients likely to have poor PFS and OS than either FLIPI, FLIPI‐2 or PRIMA‐PI. In contrast to existing clinical models, the prognostic value of the FLEX score was observed consistently across chemotherapy backbones and anti‐CD20 antibody treatment arms.

## CONFLICT OF INTERESTS

A.K., Fe.Ma.: Employed at F. Hoffmann‐La Roche Ltd.

A.G.: Advisory boards for MSD, AbbVie, Takeda and Janssen.

C.B.: Employed at Genentech, Inc. and has equity ownership interests (including stock options) in F. Hoffmann‐La Roche Ltd.

C.C.: Research funding, Celgene; honoraria and travel support, Gilead; travel support, F. Hoffmann‐La Roche Ltd.

F.M.: Previous employment, F. Hoffmann‐La Roche Ltd.

J.F.S.: Honoraria and consulting or advisory fees, AbbVie, Celgene, Genentech, Gilead, Janssen, F. Hoffmann‐La Roche Ltd and Takeda; research funding, AbbVie and Janssen; travel support, Celgene and F. Hoffmann‐La Roche Ltd.

M.H.: Research funding, F. Hoffmann‐La Roche Ltd.; membership on an entity's Board of Directors or advisory committees, Celgene, F. Hoffmann‐La Roche Ltd., Gilead, Janssen.

R.M.: Consultancy, Gilead; honoraria and speaker's bureau, F. Hoffmann‐La Roche Ltd. / Genentech, Inc.

T.N.: Employed at F. Hoffmann‐La Roche Ltd. and has equity ownership interests (including stock options) for F. Hoffmann‐La Roche.

W.H.: Consultancy, honoraria and research funding, F. Hoffmann‐La Roche Ltd., Janssen; consultancy and honoraria, Celgene, Gilead, Vector Therapeutics; research funding, Bayer.

## AUTHOR CONTRIBUTIONS

Study design: C.C., C.R.B., T.N., Fe.Ma., M.H., R.M.

Study conduct: A.G., A.K., C.C., C.R.B., T.N., Fe.Ma., J.F.S., M.H., R.M.

Data collection: A.G., T.N., W.H.

Data analysis: A.K., C.C., C.R.B., T.N., Fe.Ma., W.H. F.M.

Data interpretation: A.K., A.G., C.C., C.R.B., T.N., Fe.Ma., F.M., J.F.S., W.H.

Manuscript writing: Fe.Ma., F.M.

Manuscript review: All authors.

## Supporting information


**Appendix S1.** Supplementary Material.

## Data Availability

Qualified researchers may request access to individual patient‐level data through the clinical study data request platform: (https://vivli.org/). Further details on Roche's criteria for eligible studies are available here: (https://vivli.org/members/ourmembers/). For further details on Roche's Global Policy on the Sharing of Clinical Information and how to request access to related clinical study documents, see here: https://www.roche.com/research_and_development/who_we_are_how_we_work/clinical_trials/our_commitment_to_data_sharing.htm.
