# Supplementary material for: Follicular Lymphoma Evaluation Index (FLEX): A new clinical prognostic model that is superior to existing risk scores for predicting progression‐free survival and early treatment failure after frontline immunochemotherapy
Source: Am J Hematol. 2020 Sep 16;95(12):1503–10. doi: 10.1002/ajh.25973 (PMC7756469; doi:10.1002/ajh.25973)
Supplement: Supplementary file 1 — Appendix S1. Supplementary Material. [file AJH-95-1503-s001.docx]

**Follicular Lymphoma Evaluation Index (FLEX): a new clinical prognostic model that is superior to existing risk scores for predicting progression-free survival and early treatment failure after frontline immunochemotherapy**

**SUPPLEMENTARY MATERIAL**

**Table S1**  Baseline variables considered for inclusion in the FLEX score

|  | Variables considered | Selected/score | HR  (95% CI) | *P* value |
| --- | --- | --- | --- | --- |
| 1 | Chemotherapy: CVP* | Y / +1 | 1.80 (1.10–2.95) | 0.0195 |
| 2 | Sex: male | Y / +1 | 1.65 (1.30–2.09) | <0.0001 |
| 3 | SPD: >9320 mm^2^ on CT scan (top quartile) | Y / +1 | 1.62 (1.13–2.31) | 0.0085 |
| 4 | Histology grade 3a | Y / +1 | 1.48 (1.10–1.98) | 0.0085 |
| 5 | Extranodal sites >2 | Y / +1 | 1.46 (1.04–2.05) | 0.0292 |
| 6 | ECOG PS >1 | Y / +1 | 1.44 (0.84–2.49) | 0.186 |
| 7 | Antibody: rituximab* | Y / +1 | 1.36 (0.98–1.90) | 0.067 |
| 8 | Hemoglobin <12 g/dL | Y / +1 | 1.35 (1.01–1.81) | 0.043 |
| 9 | β_2_ microglobulin >ULN | Y / +1 | 1.29 (0.98–1.69) | 0.069 |
| 10 | NKCC <100/µL | Y / +1 | 1.26 (0.88–1.79) | 0.205 |
| 11 | LDH >ULN | Y / +1 | 1.25 (0.97–1.61) | 0.0884 |
|  | Age >60 years | N |  |  |
|  | Ann Arbor stage >II | N |  |  |
|  | B symptoms | N |  |  |
|  | BM involvement | N |  |  |
|  | Bulky site >7 cm (nodal or extranodal) | N |  |  |
|  | Geographic area | N |  |  |

*Removed from the final model to ensure the model has greater general application

*Note:* Coefficient of the selected Cox model and components of the FLEX score are ordered by HR for progression-free survival in the training (GALLIUM) cohort. HRs shown only for variables selected

BM, bone marrow; CI, confidence interval; CT, computed tomography; CVP, cyclophosphamide, vincristine, prednisone; ECOG PS, Eastern Cooperative Oncology Group performance status; FLEX, Follicular Lymphoma Evaluation Index; HR, hazard ratio; LDH, lactate dehydrogenase; NKCC, natural killer cell count; SPD, sum of the products of lesion diameters; ULN, upper limit of normal

**Table S2**  FLEX score components

| Variables | HR | 95% CI | *P* value |
| --- | --- | --- | --- |
| Sex: male (ref.: female) | 1.65 | (1.3–2.09) | 3.36e–05 |
| SPD in 4^th^ quartile >9320 mm^2^ on CT scan | 1.62 | (1.13–2.31) | 8.51e–03 |
| Histology grade 3a | 1.48 | (1.1–1.98) | 8.49e–03 |
| Extranodal involvement >2 (ref.: ≤2) | 1.46 | (1.04–2.05) | 2.92e–02 |
| ECOG PS at baseline >1 (ref.: 0–1) | 1.44 | (0.84–2.49) | 1.86e–01 |
| Hemoglobin <12 g/dL (ref.: ≥12 g/dL) | 1.35 | (1.01–1.81) | 4.30e–02 |
| β_2_-microglobulin >ULN (ref.: low) | 1.29 | (0.98–1.69) | 6.90e–02 |
| NKCC <100/µL | 1.26 | (0.88–1.79) | 2.05e–01 |
| LDH >ULN (ref.: normal) | 1.25 | (0.97–1.61) | 8.84e–02 |

*Note:* Coefficient of the selected Cox model and components of the FLEX score are ordered by HR for progression-free survival in the training (GALLIUM) cohort

CI, confidence interval; CT, computed tomography; ECOG PS, Eastern Cooperative Oncology Group performance status; FLEX, Follicular Lymphoma Evaluation Index; HR, hazard ratio; LDH, lactate dehydrogenase; NKCC, natural killer cell count; SPD, sum of the products of lesion diameters; ULN, upper limit of normal

**Figure S1** ROC curves for POD24 events A, on GALLIUM data and B, on SABRINA data, comparing linear predictors from models with and without categorization of variables.

For GALLIUM data (used to develop the model) there seems to be no loss of information from categorizing variables. For SABRINA data (external validation) some loss of discrimination ability seems to appear only towards low values of the linear predictor, which is not of concern as the interest is in high‑risk patients identification

| 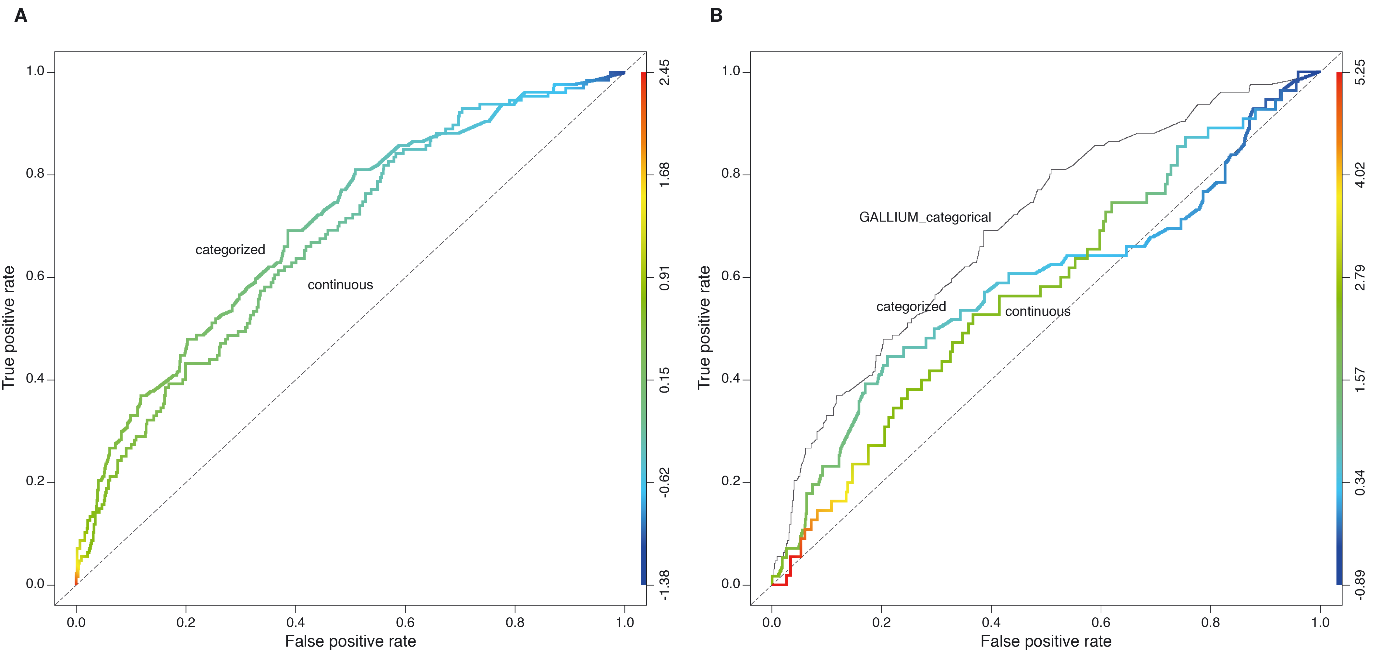 |
| --- |

POD24, progression or death due to the disease within 24 months of first-line therapy; ROC, receiver operating characteristic

**Figure S2**  Forest plot (uni‑variable Cox model) of progression-free survival in GALLIUM for A, FLEX, B, PRIMA-PI and C, FLIPI low- or non-high-risk vs high-risk patients, overall and according to treatment regimen received

| **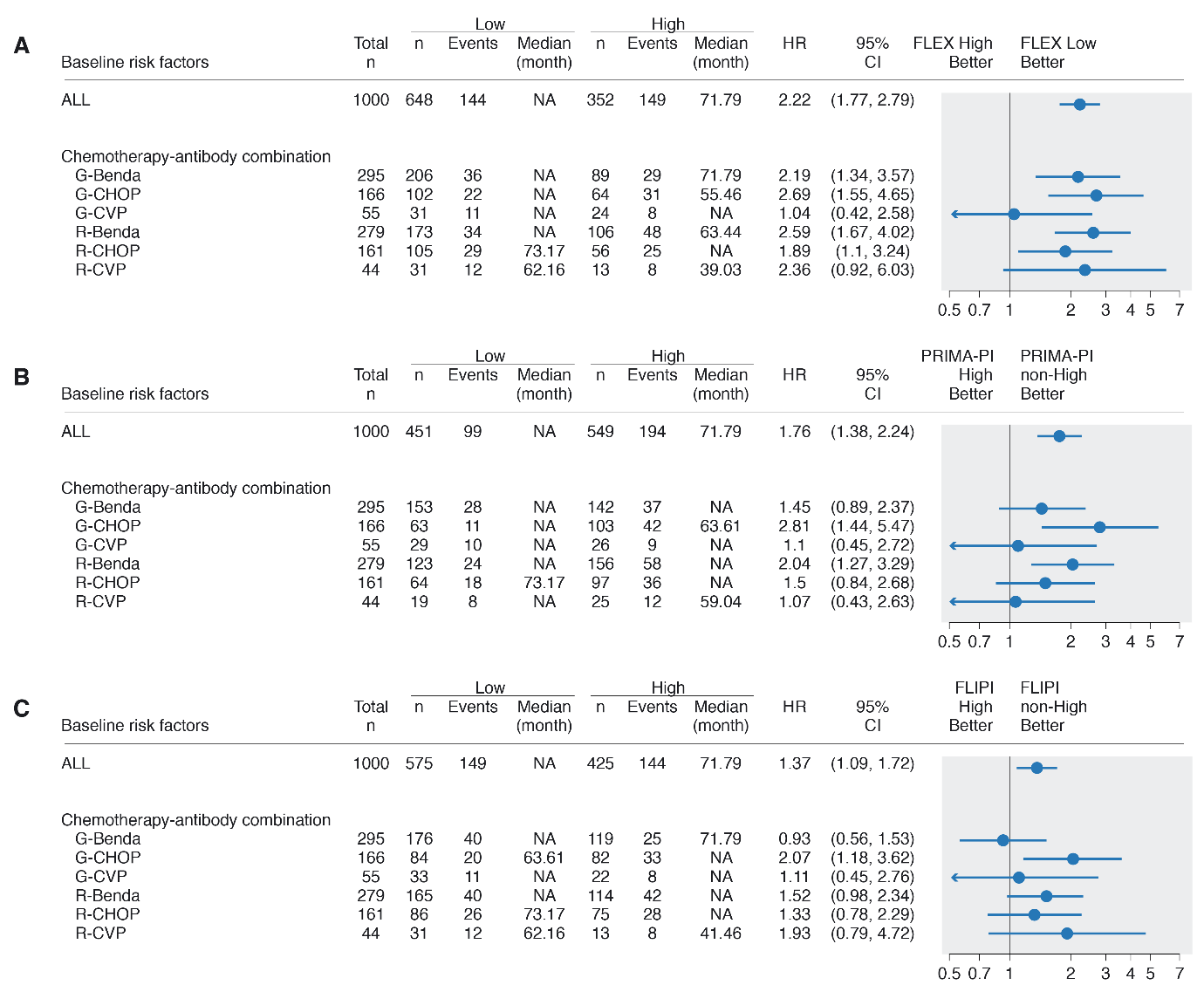** |
| --- |

Benda, bendamustine; CHOP, cyclophosphamide, doxorubicin, vincristine, prednisone; CI, confidence interval; CVP, cyclophosphamide, vincristine, prednisone; FLEX, Follicular Lymphoma Evaluation Index; FLIPI, Follicular Lymphoma International Prognostic Index; G, obinutuzumab; HR, hazard ratio; NA, not applicable; PRIMA-PI, PRIMA-Prognostic Index; R, rituximab

**Figure S3**  Forest plot (uni‑variable Cox model) of progression-free survival in GALLIUM for A, bendamustine- vs CHOP/CVP-treated patients and B, rituximab- vs obinutuzumab-treated patients, overall and according to the nine components of the FLEX score

| 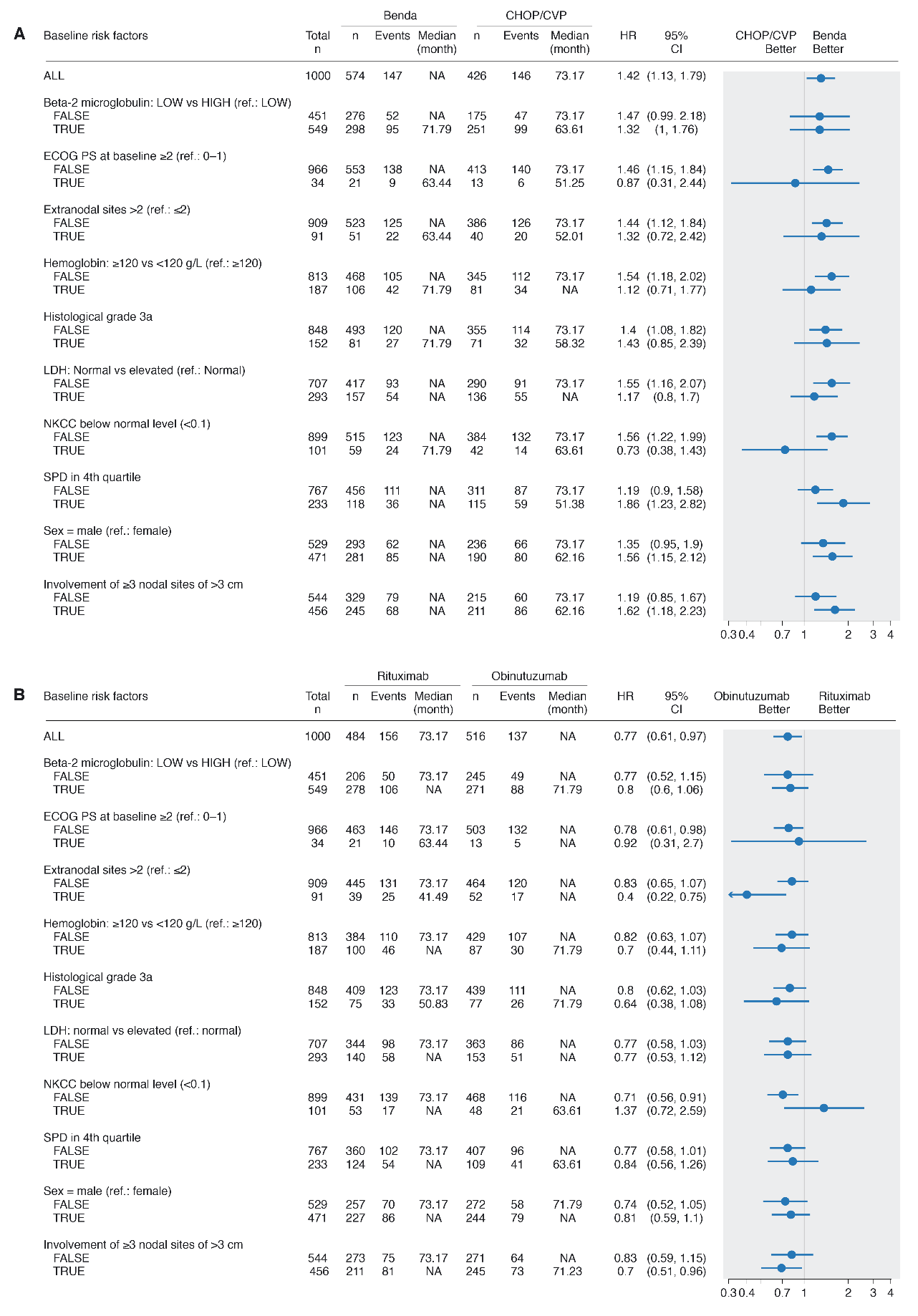 |
| --- |

Benda, bendamustine; CHOP, cyclophosphamide, doxorubicin, vincristine, prednisone; CI, confidence interval; CVP, cyclophosphamide, vincristine, prednisone; ECOG PS, Eastern Cooperative Oncology Group performance status; FLEX, Follicular Lymphoma Evaluation Index; HR, hazard ratio; LDH, lactate dehydrogenase; NA, not applicable; NKCC, natural killer cell count; SPD, sum of the products of lesion diameters

**Figure S4**  Progression-free survival according to A, FLIPI, B, FLIPI split in three groups, C, FLIPI-2 and D, PRIMA-PI in GALLIUM and SABRINA


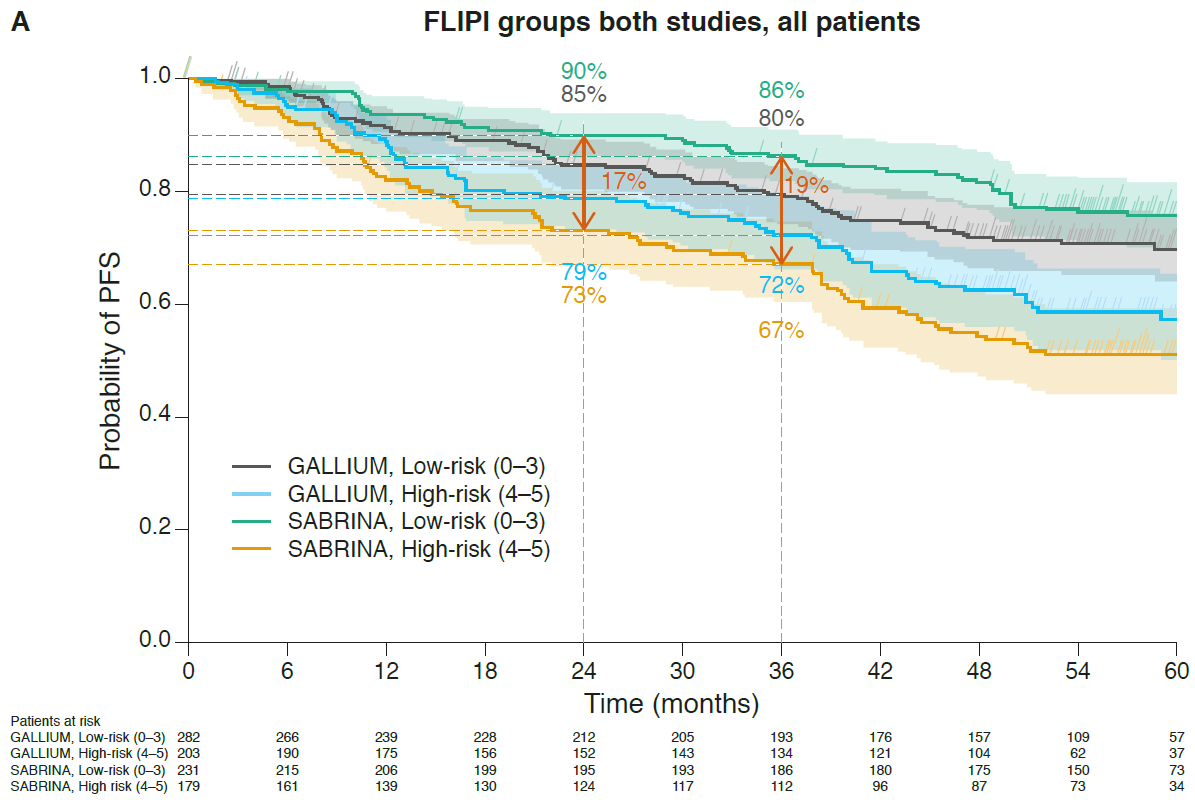


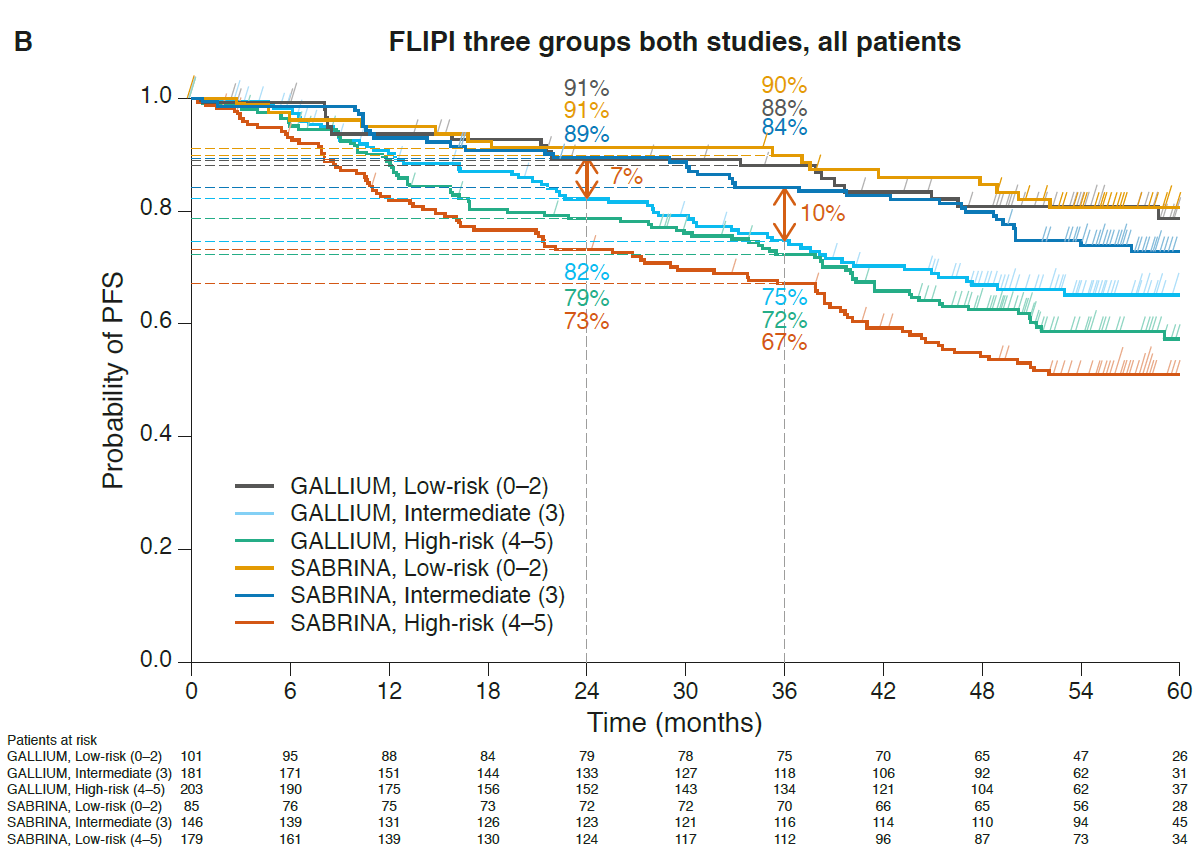


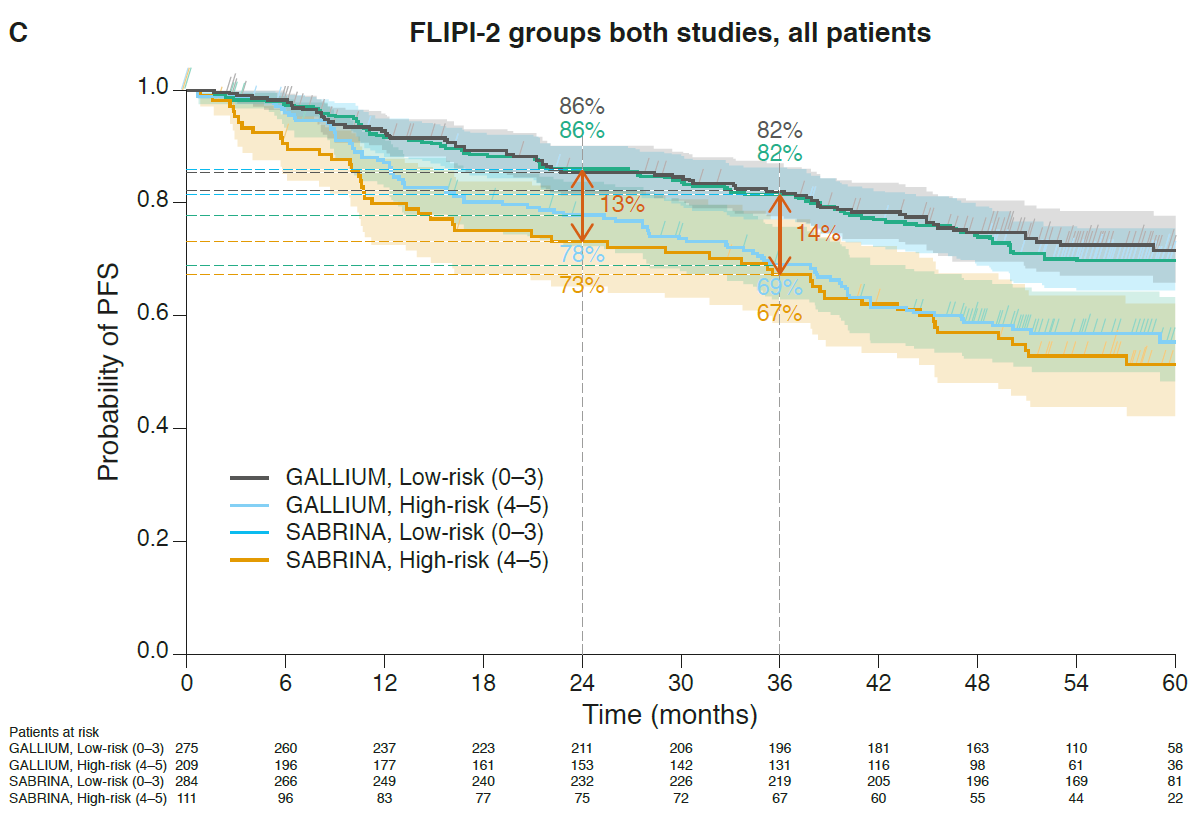


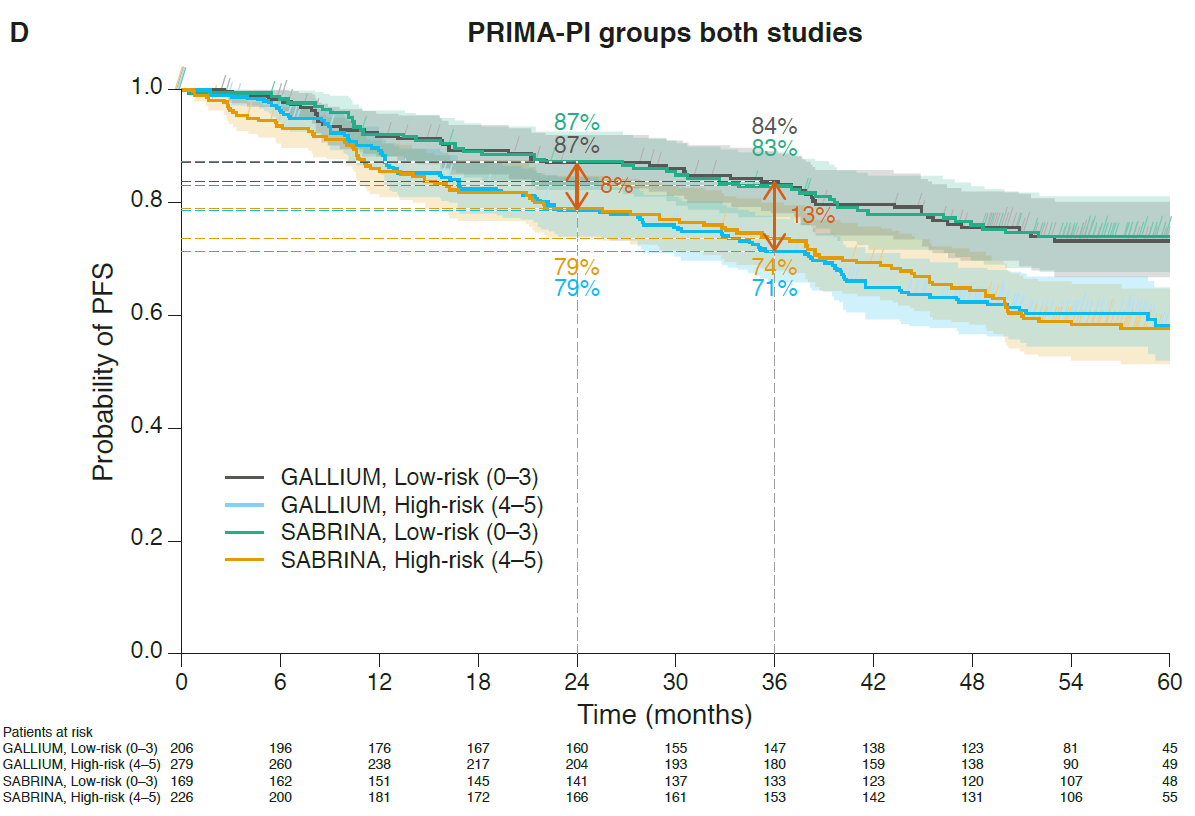


The probability of progression-free survival at 2 and 3 years and differences between the low- and high-risk categories in SABRINA are shown. FLIPI, Follicular Lymphoma International Prognostic Index; PFS, progression-free survival; PRIMA-PI, PRIMA-Prognostic Index**Figure S5**  Progression-free survival according to FLEX score split into three risk groups. Intergroup difference shown is between low risk and high risk in SABRINA


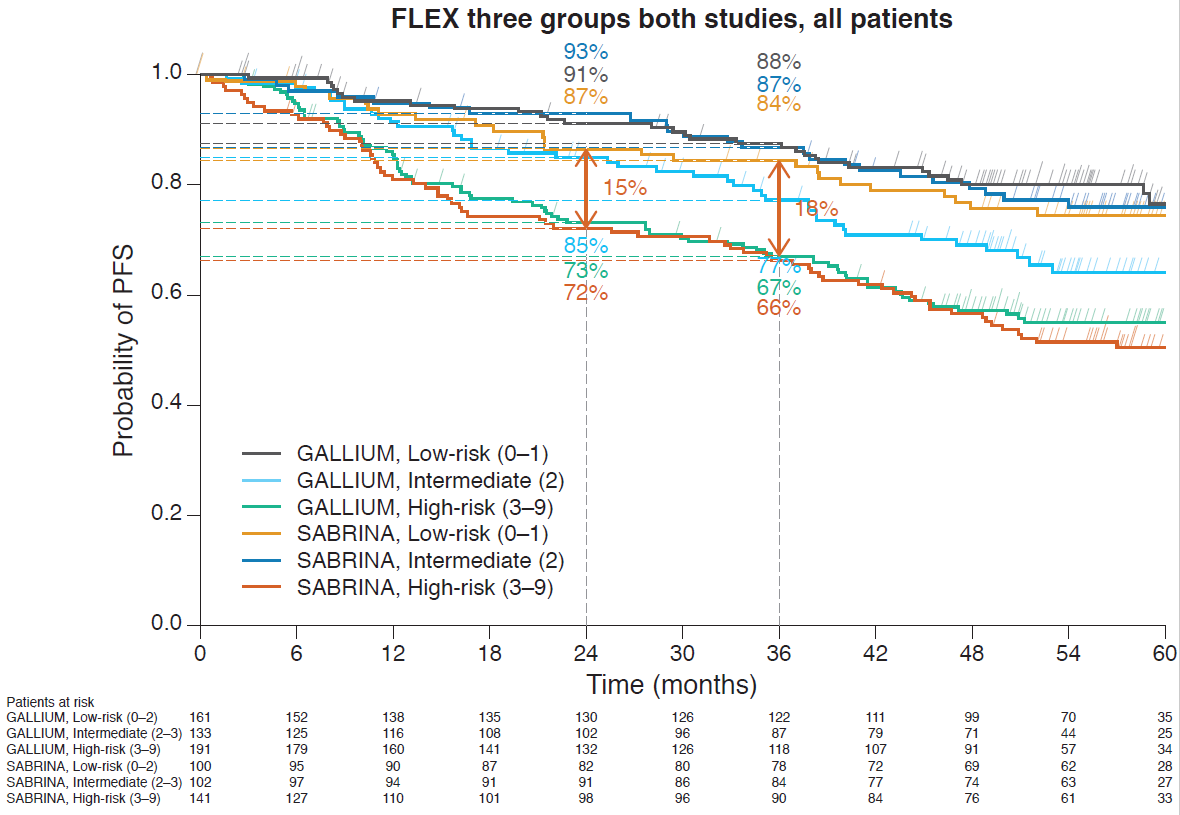


FLEX, Follicular Lymphoma Evaluation Index; PFS, progression-free survival

**Figure S6**  Forest plot (Cox model) of progression-free survival in A, GALLIUM and B, SABRINA for FLEX low- vs high-risk patients, overall and according to the nine components of the FLEX score


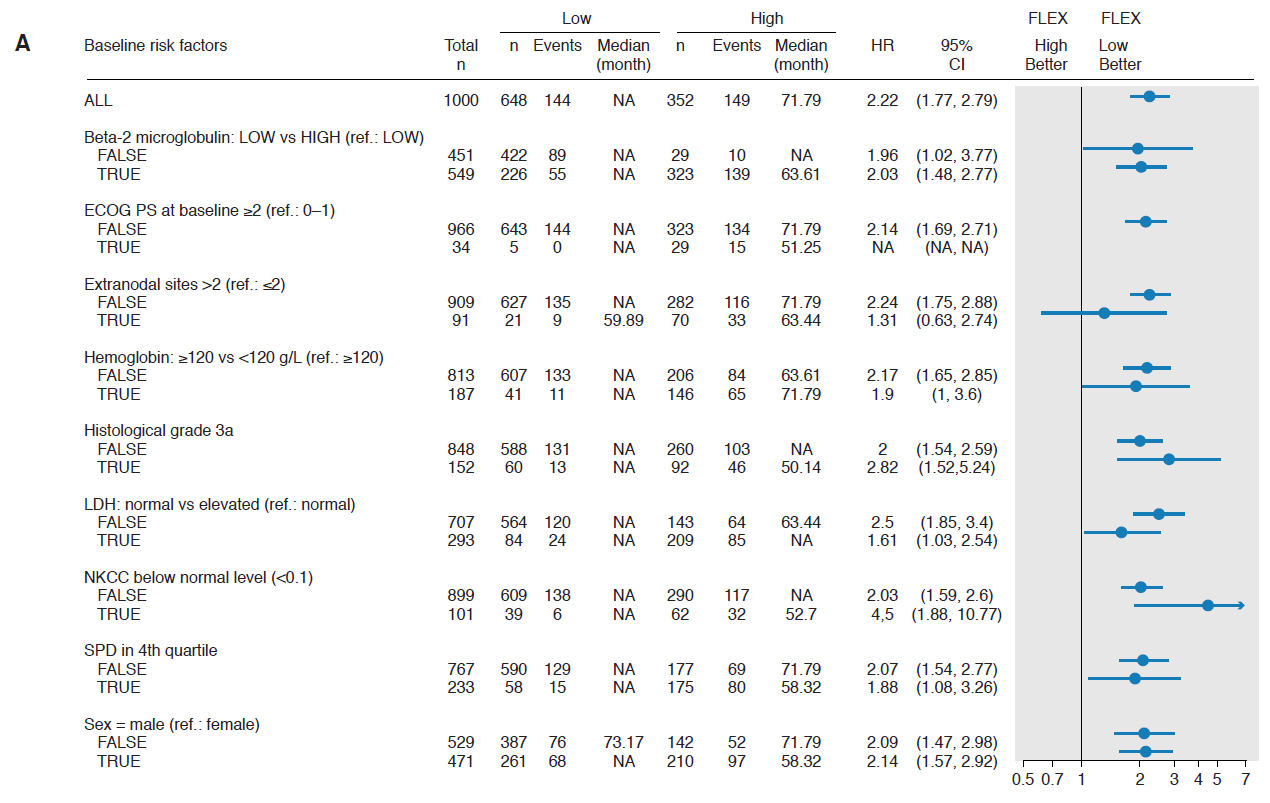


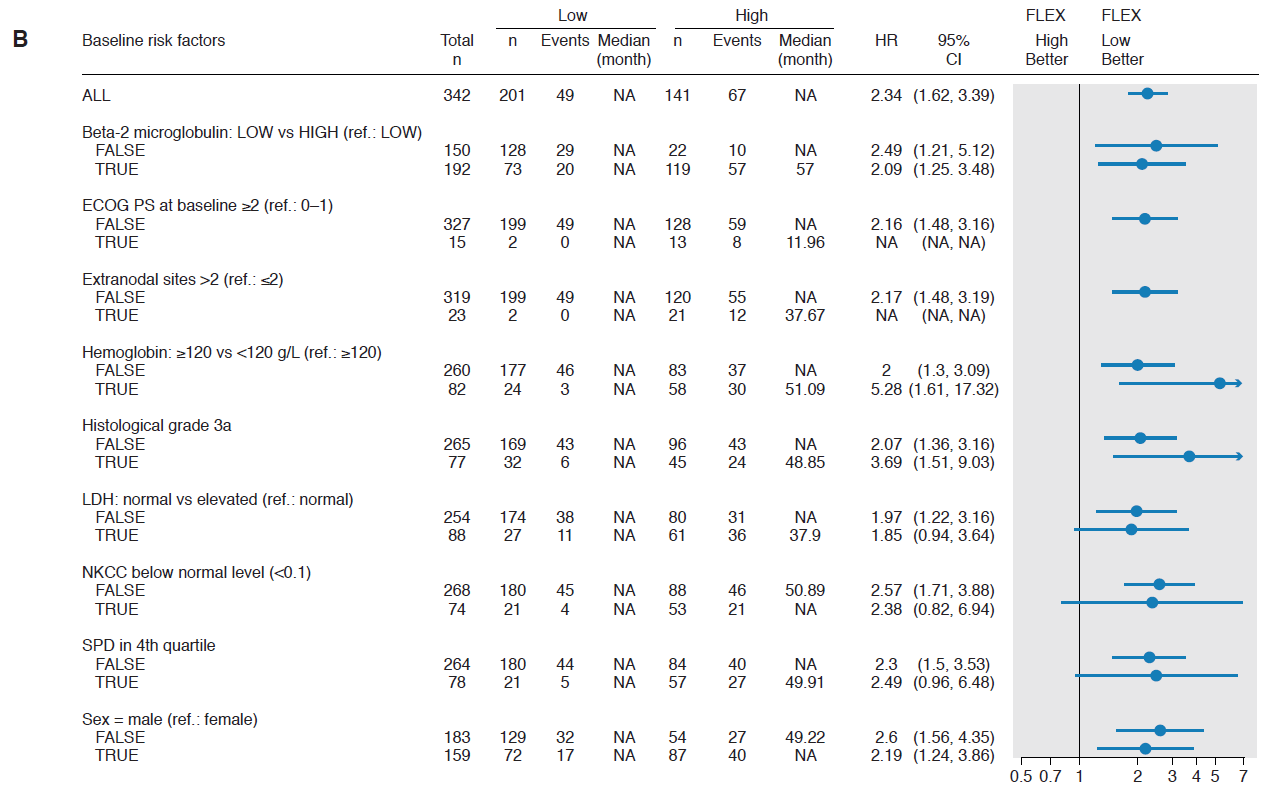


CI, confidence interval; ECOG PS, Eastern Cooperative Oncology Group performance status; FLEX, Follicular Lymphoma Evaluation Index; HR, hazard ratio; LDH, lactate dehydrogenase; NA, not applicable; NKCC, natural killer cell count; SPD, sum of the products of lesion diameters

**Figure S7** Time‑varying AUROC for PFS events on A, GALLIUM data and B, SABRINA data, comparing the different prognostic scores. Results confirm the consistent performance of FLEX in both studies, and the quite different (better) performance of FLIPI and FLIPI‑2 in SABRINA compared to GALLIUM. FLEX achieves comparable results in both studies when not dichotomized, despite not outperforming FLIPI in SABRINA


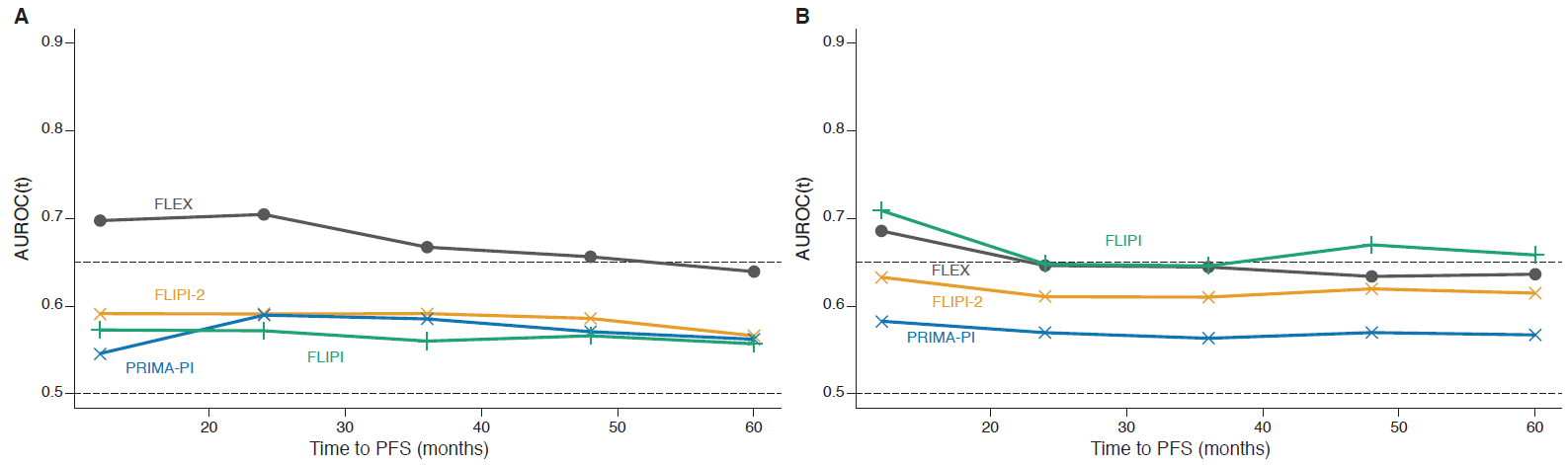


AUROC, area under the ROC curve; FLEX, Follicular Lymphoma Evaluation Index; FLIPI, Follicular Lymphoma International Prognostic Index; PFS, progression-free survival; PRIMA-PI, PRIMA-Prognostic Index
